# Environmental factors influencing DDT–DDE spatial distribution in an agricultural drainage system determined by using machine learning techniques

**DOI:** 10.1007/s10653-023-01486-y

**Published:** 2023-02-07

**Authors:** Ignacio Melendez-Pastor, Otoniel M. Lopez-Granado, Jose Navarro-Pedreño, Encarni I. Hernández, Manuel M. Jordán Vidal, Ignacio Gómez Lucas

**Affiliations:** 1 Department of Agrochemistry and Environment, Miguel Hernández University of Elche, Av. Universidad s/n, Edificio Alcudia, 03202 Elche, Alicante Spain; 2 Department of Computers Engineering, Miguel Hernández University of Elche, Av. Universidad s/n, Edificio Alcudia, 03202 Elche, Alicante Spain

**Keywords:** DDT, DDE, Spatial distribution, Soil texture, Hydrology, Random forest, Mutual information

## Abstract

The presence and persistence of pesticides in the environment are environmental problems of great concern due to the health implications for humans and wildlife. The persistence of DDT–DDE in a Mediterranean coastal plain where pesticides were widely used and were banned decades ago is the aim of this study. Different sources of analytical information from water and soil analysis and topography and geographical variables were combined with the purpose of analyzing which environmental factors are more likely to condition the spatial distribution of DDT–DDE in the drainage watercourses of the area. An approach combining machine learning techniques, such as Random Forest and Mutual Information (MI), for classifying DDT–DDE concentration levels based on other environmental predictive variables was applied. In addition, classification procedure was iteratively performed with different training/validation partitions in order to extract the most informative parameters denoted by the highest MI scores and larger accuracy assessment metrics. Distance to drain canals, soil electrical conductivity, and soil sand texture fraction were the most informative environmental variables for predicting DDT–DDE water concentration clusters.

## Introduction

Pesticide usage has helped to improve agricultural production in order to deal with global food demands and has reduced the health impact of diseases transmitted by insects such as malaria (WHO, 2011). Unfortunately, pesticide use (and abuse) is not exempt of short- and long-term effects for human and environmental health risk. In this sense, many researchers have devoted their research career to assess acute and chronic toxicological effects of pesticides in humans (Rudel et al., 2003; Walsh et al., 2017) and in other types of living organisms (Vos et al., 2000; Wauchope et al., 1992), their bioaccumulation through trophic chains (Storelli et al., 2009), and persistence in the environment (Relyea, 2009; Walsh et al., 2017).

One of the pesticides that focused the attention of the scientific community is dichlorodiphenyltrichloroethane (DDT) and its metabolites and derivatives such as dichlorodiphenyldichloroe-thylene (DDE). DDT (CAS No: 50-29-3) is a very stable, lipophilic, and persistent compound (UNEP, 2018). It has been extensively used to control malaria, typhus, and other vector-borne diseases (Van Den Berg et al., 2017). Due to its persistence, its residues can be found globally (Turusov et al., 2002). Fortunately, scientific knowledge of DDT effects on the environment has motivated the adoption of regulations and agreements to control its production and use (Li, 2018; Villaverde et al., 2016). One of the most significant agreements to minimize the environmental impacts of DDT is the Stockholm Convention on Persistent Organic Pollutants (POP). In this voluntary agreement, signatory countries have the commitment of adopting pertinent measurements for restricting its use (many countries have banned DDT usage) and production (UNEP, 2018).

Spain incorporated the Stockholm Convention on Persistent Organic Pollutants in 2001 and the last updated is from 2019 (MITECO, 2019). However, DDT (and derivatives) is more frequently detected in the hydrosphere, edaphosphere, and living organisms, even in regions like European Union whose commercialization was banned decades ago (Stemmler & Lammel, 2009; Turusov et al., 2002; Villaverde et al., 2016). This fact may suggest that if an apparently not used pesticide is detectable in the environment (e.g., drainage waters in an agricultural irrigation area), a potential reservoir remains in the soil and sediments of an specific area (Albaiges et al., 1987; Chen et al., 2020; López-Flores et al., 2003; Maillard & Imfeld, 2014) that is still emerging into surface and groundwater, potentially entering into trophic chains.

Another important issue is related to the spatial distribution of pesticides on the environment. They do not have to present a homogeneous spatial pattern. In fact, they were specially used at specific locations (e.g., waterbodies where mosquitos develop their reproductive cycle) with the aim of maximizing its effects and reducing the cost of the application. In this sense, pesticide pollution should be understood as a diffuse pollution problem, with potential presence of pesticides (and metabolites such as DDE respect to DDT) at different environmental compartments (surface waters, groundwater, sediments, soils, etc.), including areas where higher concentrations are plausible. In these areas with higher levels of pesticides, the contamination should be taken into consideration for potentially harmful effects and may require a comprehensive spatial analysis that may help to improve our environmental monitoring and land management (Chen et al., 2021).

This study focusses on the analysis of the spatial distribution of DDT–DDE in a coastal floodplain located between the Vinalopó and Segura rivers (southeast Spain), with a dense irrigation system and land usage, close to protected wetlands included in the RAMSAR sites of wetlands of global importance and the Natura 2000 network of threatened species and natural habitats of the European Union. It is well known that this area had endemic malaria until the 1960s and DDT was used to combat *Anopheles* mosquitoes (Bueno & Jiménez, 2008). A previous research allowed the identification of clusters of high or low concentrations of DDT–DDE in this area (Melendez-Pastor et al., 2021). However, the most relevant factors that may explain its spatial distribution are uncertain and require further research.

The objective of this work was to develop a methodology for the identification of major environmental factors based on water quality parameters, soil properties, topographic variables, and geographical position that may explain the spatial distribution of DDT–DDE in the drainage system of an agricultural Mediterranean coastal floodplain, by using different machine learning procedures.

## Material and methods

This study combines water and soil field surveys, laboratory analyses, and geostatistical and spatial analyses with machine learning classification techniques pursuing the aim of developing a methodological procedure to identify what environmental variables may explain current spatial distribution of a persistent pesticide in the environment such as DDT–DDE.

**Fig. 1:**
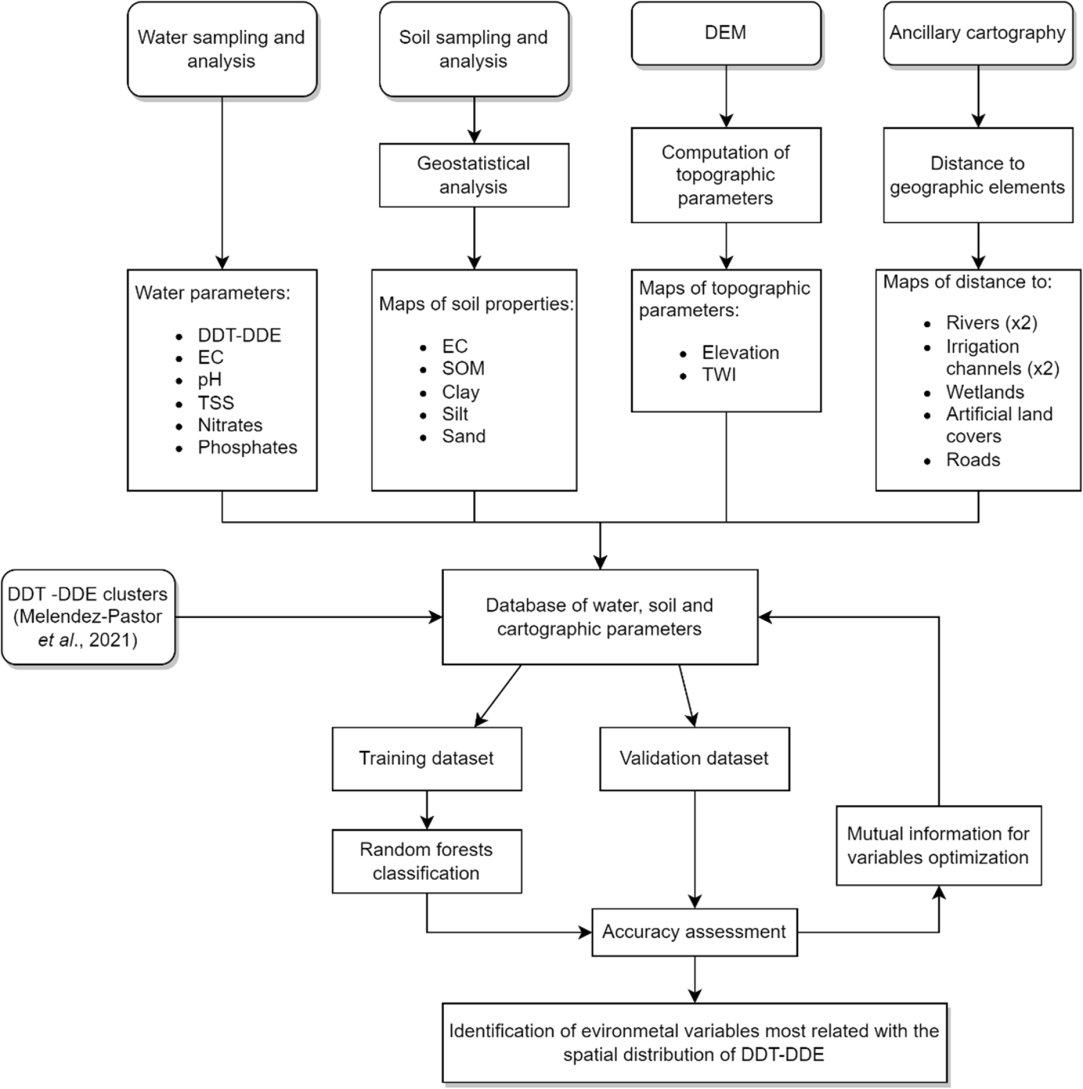
Flowchart of the methodology applied in this study

**Fig. 2:**
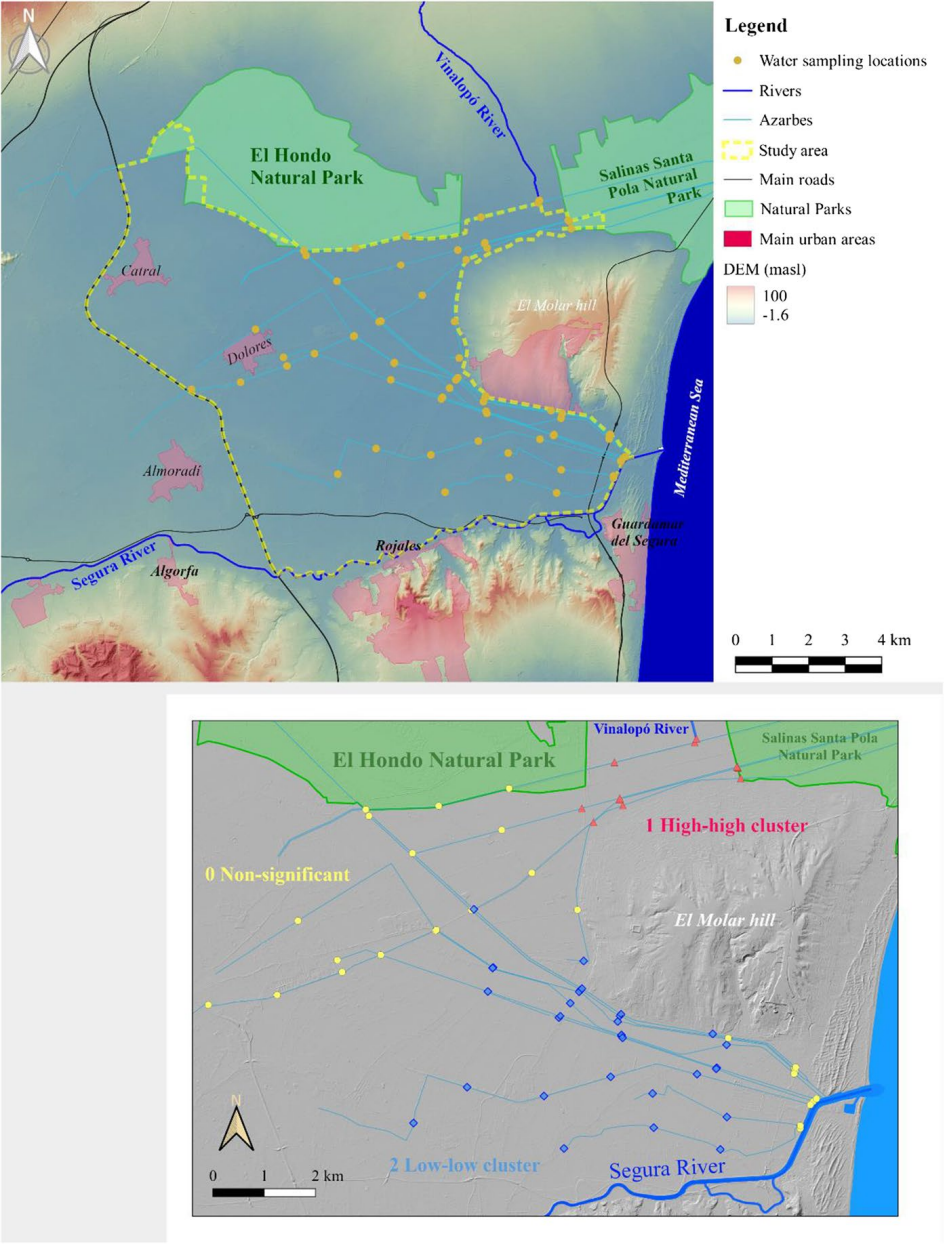
*Upper*: Location of the study area showing the drainage system and the water sampling points. *Lower*: clusters of DDT–DDE from Melendez-Pastor et al. (2021)

This methodological approach is based on five different data sources showed in the methodology flowchart (Fig. 1): (1) water samples collected across the whole study area (Fig. 2); (2) an extensive regional soil survey used to estimate and map soil properties with geostatistics; (3) a digital elevation model to obtain topographic information; (4) a set of GIS variables (i.e., hydrology, infrastructures, land cover) used to compute distance metrics; and (5) information about the clusters of DDT–DDE in the study area from a previous research (Melendez-Pastor et al., 2021). A table with the list of variables (Table 1) used in the study is included for further details. This information was used to develop a database of continuous variables, plus a discrete variable corresponding to the DDT–DDE clusters from the water analysis (i.e., 1 = high–high clusters (hotspots), 2 = low–low clusters (cold spots), and 0 = non-significant points). Then, the dataset was randomly divided into training and validation subsets, with different partition ratios (70:30, 60:40 and 50:50). Random forest (RF) was used to predict the category of the water samples (clusters of DDT–DDE) based on the list of predictive variables (Table 1). Additionally, a procedure called mutual information was used to reduce the number of predictive variables; RF was iteratively applied again with the most informative variables. Finally, accuracy assessment was computed with the confusion matrix and four accuracy metrics (i.e., overall accuracy, Cohen’s kappa, producer’s and user’s accuracy).

**Table 1:** List of variables employed in the study

Type of variable	Variables	Units	Meaning
Water	DDT–DDE	μg/L	Concentration of DDT–DDE of the water samples
	EC	mS/cm	Electrical conductivity of the water samples
	pH	–	pH of the water samples
	TSS	mg/L	Concentration of total suspended solids in the water samples
	Nitrates	mg/L	Concentration of nitrates of the water samples
	Phosphates	mg/L	Concentration of phosphates of the water samples
Soil	Soil–EC	dS/m	Map of soil electrical conductivity
	Soil–SOM	%	Maps of soil organic matter
	Soil–clay	%	Map of the soil textural fraction clay
	Soil–silt	%	Map of the soil textural fraction silt
	Soil–sand	%	Map of the soil textural fraction sand
Topography	DEM-Altitude	m	Map of terrain elevation obtained from the DEM
	DEM-TWI	m	Map of the topographic wetness index obtained from the DEM
Distance to geographic elements	Dist-V-Riv	m	Map of distance to the Vinalopó river watercourse
	Dist-S-Riv	m	Map of distance to the Segura river watercourse
	Dist-V-Aza	m	Map of distance to the drainage canals (irrigation canals) of the Vinalopó river sector
	Dist-S-Aza	m	Map of distance to the drainage canals (irrigation canals) of the Segura river sector
	Dist-Wetlands	m	Map of distance to wetland areas obtained from Corine Land Cover 2018
	Dist-Artificial	m	Map of distance to artificial areas obtained from Corine Land Cover 2018
	Dist-Roads	m	Map of distance to main roads obtained from the Valencian Cartographic Institute

Its typology, abbreviation and explanation are included

### Description of the study area

The area is located in the southeast of Spain (province of Alicante), in a coastal floodplain that, centuries ago, was a large coastal marsh and a lagoon—Elche lagoon—(Box Amorós, 2004). Due to the progressive implementation of drainage infrastructures (especially in the eighteenth century), the current landscape is a mixture of irrigated agricultural areas and wetlands, with scattered urban settlements.

The area where this research is focused comprises an extension of 89 km^2^, and its delimitation was done according to natural and artificial features in order to enclose the large coastal plain area. This is located over quaternary sediments, mainly transported by two rivers (Segura River in the south and Vinalopó River in the north), whose flow converged into the ancient lagoon. That lagoon was separated from the sea by a sand dune barrier that is still present. The relief of the study area is very flat (Fig. 2), with a median ground elevation of 3.2 m.a.s.l. and average slope below 1%. Respect to the soils, the most frequent class are calcaric fluvisols. Additionally, other well-represented classes are Solonchaks (due to the high soil salinity and aridity of the area) and anthrosols (by profoundly modifications through many years of term cultivation) (Bas-Niñerola et al., 2017; IUSS Working Group WRB, 2014).

The climate class corresponds to *BSh* (hot semi-arid climate) based on the Köppen–Geiger classification. It is characterized by an annual average temperature about 18 °C and less than 300 mm of average precipitation but with very acute inter-annual variations (AEMET-IMP, 2011). In this sense, precipitation temporal variability promotes periods of severe drought, but contrasting with periods of intense precipitations that may provoke dangerous flood events. Climate and edaphic conditions impose some restrictions for agriculture that is possible thanks to a very intricate network of drainage canals (locally called “azarbes”) that allow draining the water, reducing groundwater level to desiccate wetlands and marshes, and increasing the arable land (Fig. 2, lower). Respect to current land uses of the study area, Corine Land Cover 2018 classification (EEA, 2017) reports that 89% of the study area is for agriculture, primarily irrigated land (69.5% of the total area), and fruit trees and berry plantations (13.2% of the total area). One of the most dramatic land cover changes in the last centuries was wetland desiccation. Nowadays, some wetland areas persisted in and around the study perimeter and were transformed into irrigation water reservoirs (also for hunting and fishing), most of them inside the current Natural Park of “El Hondo” (Box Amorós, 2004).

Respect to the configuration of the irrigation systems, two sectors could be distinguished: (1) the southern sector corresponding to the Segura River and associated canals; and (2) the northern sector corresponding to the Vinalopó river and associated canals. For both cases, irrigation canals finally converge for a single water flow into the Mediterranean Sea, the southern sector to an artificial mouth of the Segura River, and the northern sector to a mouth in the middle of the Salinas de Santa Pola saltworks.

### Water sampling and analysis

A field campaign to collect water samples was done across 15 watercourses, including the mouth of both rivers, and 13 drainage canals (azarbes). We collected 76 water samples (Fig. 2) during August 2017. A sampling pole was employed to collect water samples in the middle of the water course with the aim of minimizing the resuspension of sediments from the bottom of the river or canal. Samples were stored at 4 °C until their analysis in the laboratory.

Six water quality parameters were analyzed, namely pH, electrical conductivity (EC), total suspended solids (TSS), nitrates, phosphates, and pesticides (DDT–DDE). Standard methods (APHA-AWWA-WEF, 2012) were followed to quantify pH, EC, TSS, and nitrates, and phosphates were determined with a standardized photometric system, while pesticides were determined with an enzyme-linked immunosorbent assay (ELISA) test kit. Firstly, pH was determined with a Crison pH meter GLP21 with electrical conductivity at 25 °C with a Crison conductometer GLP31. Then, water samples were filtered in order to determine TSS and to obtain filtered water for subsequent analyses. We employed 0.45 µm of pore diameter glass microfiber filters (Whatman-Cytiva, Marlborough, MA, USA). The filters with retained particles were dried in an oven (105 ◦C) until constant weight. Total suspended solids were determined by gravimetry. The second derivative method was employed to quantify nitrates in the water samples. This determination was done with a PG Instruments T80 UV/VIS Spectrometer (PG Instruments Limited, Alma Park, UK). Phosphate determination was done with an Orion AQUAfast AQ3700 Colorimeter (Thermo Scientific Inc., Waltham MA, USA) and its ortho-phosphate powder and reaction tube kit (reference ACR095). This test, as an Environmental Protection Agency (EPA)-approved method, measures in the range of 0.06–5.0 mg/L.

Respect to the pesticide analysis, we employed a microtiter plate ELISA test kit for DDT–DDE in water samples (Eurofins Abraxis, Warminster, PA USA). The test jointly quantifies DDT and the metabolite DDE, and the obtained value is the sum of DDT plus DDE. Concentrations of the samples were determined using the standard curve run with each test (standards: 0.625; 1.25; 2.5; 5.0; 10.0; 25.0 ppb). The upper and lower detection limits were 25.0 and 0.625, ppb, respectively. Regarding the sensitivity of the test, this DDE/DDT assay has an estimated minimum detectable concentration, based on 90% B/B_0_ of 0.4 ng/mL (Eurofins Abraxis product code 540,041). Finally, a microplate reader was employed to quantify the results from the DDT–DDE ELISA test kit (HEALES MB-580, Shenzhen Huisong Technology Development Co. Ltd., Shenzhen, China). Only 69 water samples had DDT–DDE values within the detectability range of the ELISA test (seven samples had DDT–DDE values below the lower detection limit), and further analyses were done with the water samples where DDT–DDE was detected.

### Soil sampling and analysis

We employed an extensive field survey in the region, including 130 topsoil samples (upper 10 cm) randomly distributed through the study area, air dried at room temperature, and sieved (2 mm) to separate the soil fine fraction to be analyzed (Bas-Niñerola et al., 2017). In this study, we employed six soil parameters, namely electrical conductivity, pH, soil organic matter (SOM), and the three texture classes (clay, silt, and sand fractions). pH (1:2.5 w/v in water extraction) was determined with a Crison pH meter GLP21 with electrical conductivity at 1:5 w/v in water extraction with a Crison conductometer GLP31. Soil organic matter (SOM) was determined by wet chemical oxidation with potassium dichromate oxidation based on Walkley–Black method (Nelson & Sommers, 1982; Walkley & Black, 1934). Texture determination (clay, silt, and sand fractions) was based on the Bouyoucos method (Gee & Bauder, 1986).

### Soil property mapping with geostatistics

Geostatistical analyses were used to obtain maps of the soil properties for the study area. The purpose of the geostatistical analysis was the estimation of soil parameters at unsampled locations (e.g., around the locations of the water samples). It implies the computation and modeling of the variogram of a regionalized variable (Matheron, 1962), such as our soil properties. Experimental variograms were modeled on the R language. We employed the *GSTAT* package (Pebesma & Wesseling, 1998) for modeling the experimental variograms of our soil properties. Based on our previous experience on mapping soil properties with geostatistics in the study area (Bas-Niñerola et al., 2017; Juan et al., 2011; Navarro-Pedreño et al., 2007), we adopted the following procedure:

- Our soil properties’ dataset (*n* = 130) was divided into two independent randomly selected subsets, one for calibration (*n* = 100) of the exploratory analysis and geostatistical modelling, and another for validation (*n* = 30) of our soil property mapping estimations.
- Isotropic variograms were computed for each variable (Lark et al., 2006) and variogram fitting was done with the Cressie’s robust estimator method (Cressie, 1993). First- and second-order trend surfaces were computed for each soil property and variogram fitting with the residuals was also assessed (Bas-Niñerola et al., 2017).
- Model fitting error (R^2^ and RMSE) was evaluated to iteratively guide the selection of the most plausible theoretical variogram. Soil property mapping was done with the better experimental variogram models.
- Soil property maps were obtained by estimating such variables with the ordinary kriging (OK) estimator. Ordinary kriging is largely used for soil sciences applications (Lark, 2012; Webster & Oliver, 2007). Ordinary kriging assumes a constant, but unknown local mean and the analysis proceeds on the assumption of intrinsic stationarity requirement (Lark, 2012).
- Finally, map accuracy was assessed with the independent validation dataset by computing several metrics, including R^2^ and the mean absolute error (MAE).

### Topographic features and ancillary cartography

A digital elevation model (DEM) obtained from the Spanish National Geographic Institute (IGN) was used. This model is publicly available (www.ign.es), has been obtained by interpolation from the ground class of LIDAR flights of the first coverage of the National Plan for Aerial Orthophotography (PNOA), and has a spatial resolution of 5 m. The elevation (DEM) was one of the explanatory variables used to understand the spatial distribution of DDT–DDE. Topographic wetness index (TWI) was computed from the DEM as another explanatory variable. The TWI was developed by Beven and Kirkby (1979) and is computed as follows:

$${\text{TWI}} = \ln \left( {\frac{a}{\tan \beta }} \right)$$

where *a* is the local upslope area draining through a certain point per unit contour length and tan* β* is the local slope. The TWI has been used to study spatial scale effects on hydrological processes, to identify hydrological flow paths for geochemical modelling, and to characterize biological processes (Sørensen et al., 2006).

We also used the following additional geographical variables as explanatory variables:

1. *Rivers courses:* Digitalized (vector features) from the most recent aerial orthophotography (2021) obtained from National Plan for Aerial Orthophotography (PNOA) with a pixel resolution of 15 cm.
2. *Irrigation canals:* Digitalized (vector features) from the most recent images (2021) obtained from National Plan for Aerial Orthophotography (PNOA) with a pixel resolution of the 15 cm.
3. *Wetland areas:* Obtained from the Corine Land Cover 2018 (EEA, 2017). This is included in the Corine Land Cover classes: 411 inland marshes, 421 salt marshes, 422 salines, and 521 coastal lagoons.
4. *Artificial land covers:* Obtained from the Corine Land Cover 2018 (EEA, 2017). This layer included the following Corine Land Cover classes: 111 continuous urban fabric; 112 discontinuous urban fabric; 121 industrial or commercial units; 131 mineral extraction sites; 132 dump sites; 133 construction sites; and 142 sport and leisure facilities.
5. *Main roads:* Obtained from the Valencian Cartographic Institute (ICV) and publicly available (https://icv.gva.es). This cartography had a scale 1:5000 and was updated in March 2022.

To compute these variables, vector features were converted to raster at the standard resolution of the study (30 m) and after that, distance-to-features maps were computed.

### Random forest classification

Values from the cartographic products (i.e., soil parameters, topographic features, and distance maps) were extracted from the pixels corresponding to the coordinates of the water samples. A database with all the analytical parameters (*n* = 69) was used for subsequent analyses. Based on our previous research (Melendez-Pastor et al., 2021), the database included the identification of the points as high–high clusters (hotspots) of DDT–DDE (category value = 1; *n* = 11), low–low clusters (cold spots) of DDT–DDE (category value = 2; *n* = 21), or non-significant clusters of DDT–DDE (category value = 0; *n* = 37).

We also explored the influence of three different partitions of the dataset on the accuracy of the classification. The full database was randomly divided with a fixed seed into training and validation subsets, with different ratios (training/validation): 70:30, 60:40, and 50:50. Then, samples were classified according to the categories of DDT–DDE cluster of the training dataset, and its accuracy was assessed with the independent validation subset. The machine learning classification technique chosen for this task was Random Forest (Ho, 1995).

The Random Forest (RF) method was firstly introduced by Breiman (2001). This method is based on the concept of ensemble building of decision trees. It is a non-parametric and efficient classification technique that provides high classification accuracy for various different applications. RF uses feature randomness and bagging when building each individual decision tree to produce an independent forest of trees. The prediction by this committee is more accurate than that of any individual tree and is robust against overfitting. RF provides all the benefits of a decision tree with the added efficiency of using more than one model (Liaw & Wiener, 2002). Opposite to standard decision trees, where each node is split using the best split among all parameters, in RF, each node is split using the best split among a subset of parameters that is randomly chosen (Breiman, 2001).

The construction of RF was determined by four parameters (Breiman, 2001; Dietterich, 2000): (1) the number of samples in the random subset (*maximal depth*); (2) the number of trees in the forest to be included; (3) *Gini impurity index* that is determined by deducting the sum of squared of probabilities of each class from one and indicates the amount of probability of a specific feature that is classified incorrectly when selected randomly; and (4) the maximum number of features (*max_features*) the RF is allowed to try in an individual tree, that in our case was the square root of the number of input features. In this study, we have used the default values with a number of trees of 100 and a maximal depth of 10 (no experiment used a depth greater than 7 to generate the RF). All the experiments were conducted with Python version 3.9.7, using the *sklearn* package version 0.24.2, with a computer running Windows 10 as operating system, and following hardware specifications Intel(R) Core(TM) i7-6800K CPU @ 3.40 GHz, 64 GB RAM memory. The computation time of the random forests was 102 ms (± 2 ms).

### Mutual information

Feature selection methods have been widely used to reduce computation time, to improve prediction performance, and also to provide a better understanding of the data in machine learning or pattern recognition applications. After training and evaluating the RF classification model using the whole input variables, we used Mutual Information (MI) as feature selection for the machine learning input variables. MI is a measure of the amount of information one random variable contains about another (Cover & Thomas, 2006). Based on the concept of Shannon entropy (Shannon, 1948), MI is a special case of a more general quantity called relative entropy, which is a measure of the distance between two probability distributions (Cover & Thomas, 2006). For two random variables *X* and *Y* with a joint probability mass function *p*(*x*, *y*) and marginal probability mass functions *p*(*x*) and *p*(*y*), the mutual information *I*(*X*; *Y*) is the relative entropy between the joint distribution and the product distribution *p*(*x*)*p*(*y*) (Cover & Thomas, 2006):

$$I\left( {X;Y} \right) = \mathop \sum \limits_{x \in X} \;\mathop \sum \limits_{y \in Y} \;p\left( {x,\;y} \right)\;\log \frac{{p\left( {x,\;y} \right)}}{p\left( x \right)\;p\left( y \right)}.$$

The continuous form of MI considers probability densities (${\hat{f}}$) of the continuous variables (such us in our study) and is defined as follows (Steuer et al., 2002):

$$\hat I\left( {X;Y} \right) = \int\limits_x {\int\limits_y {\hat f\left( {x,y} \right)\;\log \frac{{\hat f\left( {x,y} \right)}}{\hat f\left( x \right)\;\hat f\left( y \right)}\;{\text{d}}x\;{\text{d}}y} } .$$

MI contains information about all linear and non-linear dependencies. MI is in several ways a perfect statistic for measuring the degree of relatedness between datasets (Ross, 2014).

### Accuracy assessment

Accuracy assessment of the classification was evaluated with the confusion matrix and four metrics based on it. The confusion (or error) matrix is a form of contingency table showing the differences between the true and predicted classes for a set of labeled items. Although the error matrix shows all of the information about the classifier’s performance, more meaningful measures can be extracted from it (i.e., accuracy metrics) to illustrate a performance criterion (Bradley, 1997). In addition, the error matrix is an effective method for evaluating both the error of inclusion (commission error) and the error of exclusion (omission error) present in the classification as well as the overall accuracy (% correct matches) (Congalton et al., 1983).

*Overall accuracy* was computed by dividing the total number of correctly classified items (i.e., the sum of the figures along the major diagonal) by the total number of features in the confusion matrix. *Producer’s accuracy* (omission error) results by dividing the number of correctly classified pixels in each category by the number of training samples used for that category. This metric indicates how well training samples of the given category area are classified. *User’s accuracy* (commission error) is computed by dividing the number of correctly classified pixels in each category by the total number of pixels that were classified in that category. This metric indicates the probability that an item classified into a given category actually represents that category (Lillesand et al., 2004).

The kappa coefficient developed by Cohen (Cohen, 1960) is a measure of overall agreement for nominal scales based on the difference between the actual agreement of the classification (i.e., agreement between the computer classification and reference data indicated by the diagonal figures) and the chance agreement, which is indicated by the product of the row and column marginal (Congalton et al., 1983). This measure of agreement (also called KHAT) is calculated by:

$$\hat K = \frac{{\mathop \sum \nolimits_{i = 1}^r {x_{ii}} - \mathop \sum \nolimits_{i = 1}^r \left( {{x_{i + }}*{x_{ + i}}} \right)}}{{{N^2} - \mathop \sum \nolimits_{i = 1}^r \left( {{x_{i + }}*{x_{ + i}}} \right)}}$$

where *r* is the number of rows in the matrix, *x*_*ii*_ is the number of observations in row *i* and column *i* (i.e., the *i*th diagonal element), *x*_*i*+_ and *x*_+*i*_ are the marginal totals of row *i* and column *i*, respectively, and *N* is the total number of observations (Bishop et al., 2007). Kappa coefficient is computed for each matrix and is a measure of how well the classification agrees with the reference value (Congalton et al., 1983). Values closer to 1 indicate higher overall accuracy while close to 0 indicate no agreement (Tang et al., 2015).

## Results and discussion

The main characteristics of the variables used for predicting the clusters of DDT–DDE with the RF classification are summarized in Table 2. The average DDT–DDE concentration was 1.45 μg/L (SD = 0.87 μg/L), with a minimum and maximum concentrations of 0.70 μg/L and 6.53 μg/L respectively. Water quality parameters showed low variability for pH (average pH = 7.9) and high electrical conductivity values (average EC = 5.7 mS/cm). Generally, electrical conductivity of the water samples obtained in the drainage canals of the Vinalopó river irrigation system exhibited higher electrical conductivity values (Melendez-Pastor et al., 2021). The average total suspended solids was 67.5 mg/L with a maximum of 538.8 mg/L. Nitrate concentration values ranged from 0.67 to 48.70 mg/L, with higher values for the drainage canals of the Segura river irrigation system. Phosphate average concentration was 0.97 mg/L, with a maximum of 12.78 mg/L for an azarbe of the Vinalopó river.

**Table 2:** Descriptive statistics of the variables employed in the study (*n* = 69)

Variables	Units	Minimum	Maximum	Mean	SD
DDT–DDE	μg/L	0.70	6.53	1.45	0.87
EC	mS/cm	2.30	18.16	5.70	3.13
pH	–	7.4	8.4	7.9	0.2
TSS	mg/L	2.40	538.80	67.52	74.53
Nitrates	mg/L	0.67	48.70	22.14	12.95
Phosphates	mg/L	0.00	12.78	0.97	1.62
Soil–EC	dS/m	0.48	3.09	1.55	0.55
Soil–SOM	%	1.8	2.8	2.4	0.2
Soil–clay	%	16.4	46.9	32.2	7.3
Soil–silt	%	24.5	73.6	42.4	8.4
Soil–sand	%	7.3	49.1	21.2	12.2
DEM-Altitude	m	0.2	5.2	2.5	1.0
DEM-TWI	m	− 5.8	6.8	1.3	3.6
Dist-V-Riv	m	0.0	10,819.7	5731.7	2295.6
Dist-S-Riv	m	84.9	8559.8	4118.0	2528.0
Dist-V-Aza	m	0.0	5275.7	1849.3	1762.0
Dist-S-Aza	m	0.0	5314.7	783.8	1592.7
Dist-Wetlands	m	0.0	5075.7	1972.7	1594.4
Dist-Artificial	m	0.0	2898.2	707.8	767.4
Dist-Roads	m	0.0	870.0	185.8	213.3

Minimum, maximum, mean, and standard deviation values area included

**Fig. 3:**
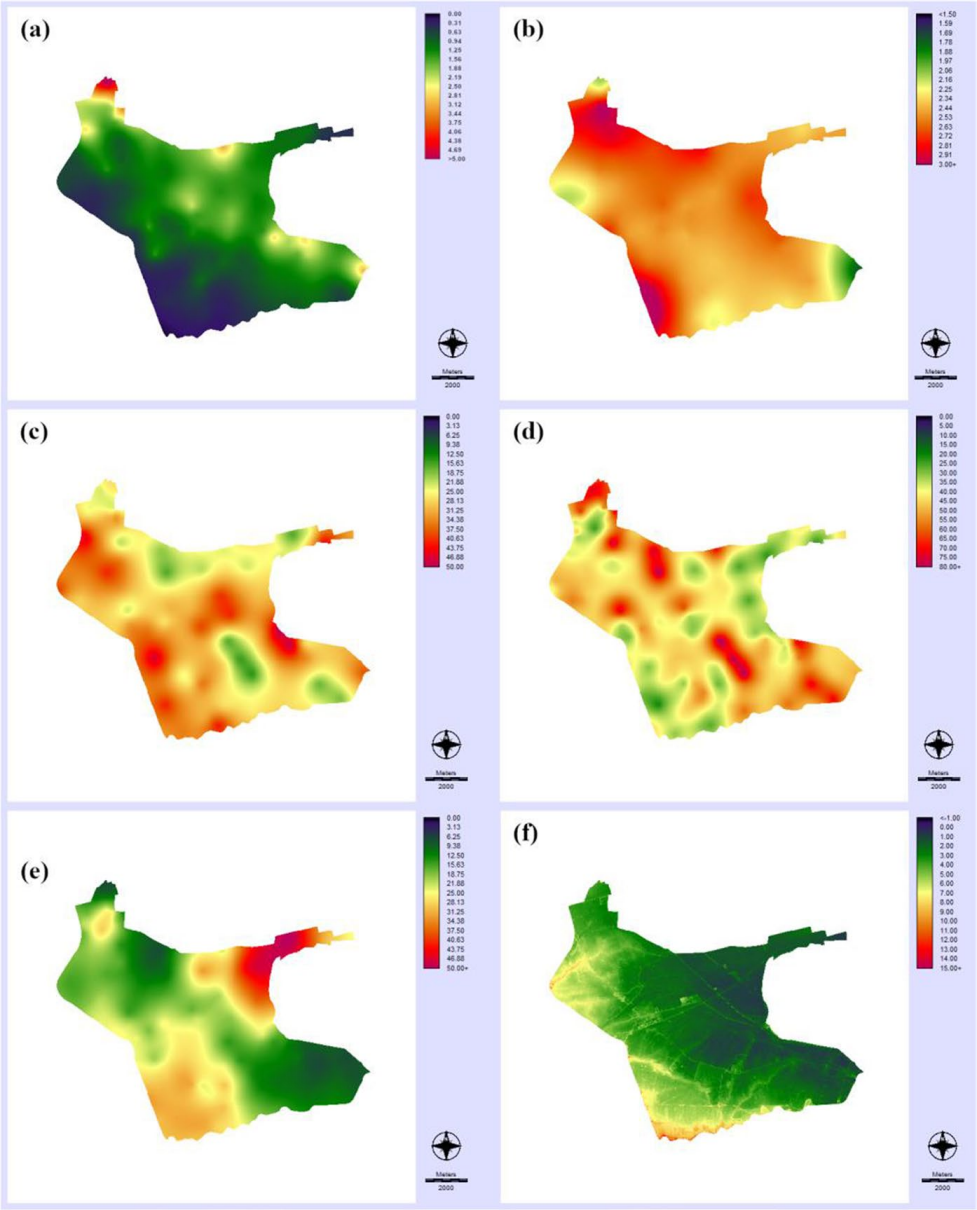
Maps of variables: **a** soil electrical conductivity (Soil-EC); **b** soil organic matter (soil–SOM); **c** soil textural fraction clay (soil–clay); **d** soil textural fraction silt (soil–silt); **e** soil textural fraction sand (soil–sand); and **f** terrain elevation (DEM–altitude)

**Fig. 4:**
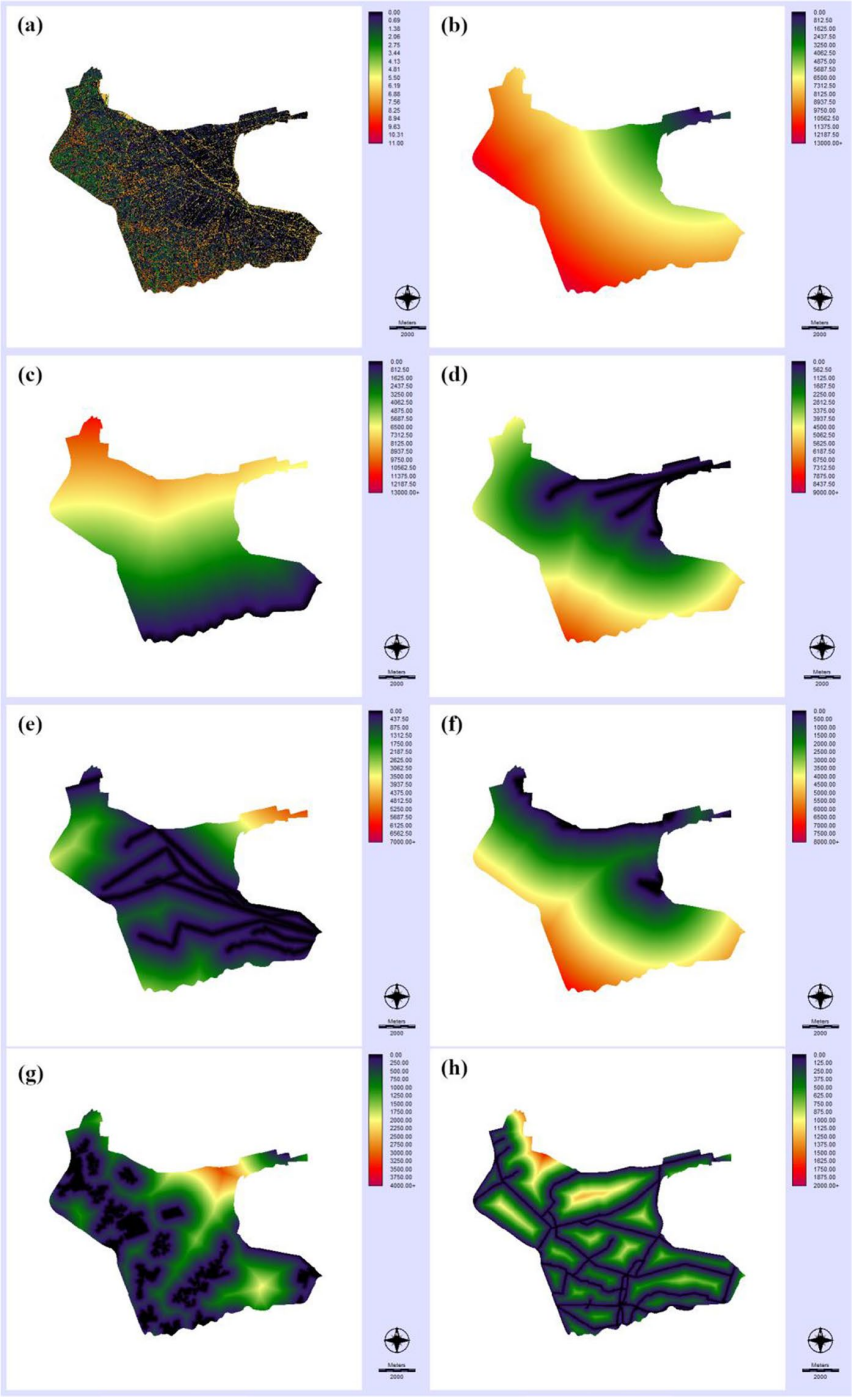
Maps of variables (continuation): **a** topographic wetness index (DEM-TWI); **b** distance to the Vinalopó river (Dist-V-Riv); **c** distance to the Segura river (Dist-S-Riv); **d** distance to the Vinalopó river’s azarbes (Dist-V-Aza); **e** distance to the Segura river’s azarbes(Dist-S-Aza); **f** distance to wetland areas (Dist-Wetlands); **g** distance to artificial areas (Dist-Artificial); and **h** distance to main roads (Dist-Roads)

The location of the water samples was used to extract information from coincident pixels of the maps of explanatory variables developed for this study (Figs. 3, 4). Soil property maps (Fig. 3a–e) were obtained with geostatistics. Variographic analyses were done with the raw soil data except for electrical conductivity. In this case, a clear north-to-south trend was observed and variographic analysis was done with the residuals after computing a second-order trend surface. Ordinary kriging was the interpolation technique employed for mapping soil properties. Soil electrical conductivity exhibited a wide range of values with a maximum of 3.08 dS/m and a minimum of 0.48 dS/m (Table 2). Soil salinity is a problem of great concern in the study area that even conditions the distribution of crops (Bas-Niñerola et al., 2017). In fact, some parts of the study area are unproductive for agriculture and are occupied by saltmarshes. The average soil organic matter content was 2.4%, with a limited spatial variability. Respect to soil texture fractions, the average values were 32.2% for clay, 42.2% for silt, and 21.2% for sand. Higher spatial variability was observed for the sand fraction (minimum of 7.3% and maximum of 73.6%), with higher values in the northeast of the study area (Fig. 3e). Accuracy assessment results of the geostatistical analyses revealed significant Pearson correlation coefficient values (*R*^2^), with a maximum for soil electrical conductivity (0.72). Mean absolute error (MAE) values were 0.40 dS/m for soil electrical conductivity, 0.50% for soil organic matter, 7.8% for clay fraction, 5.2% for silt fraction, and 8.9% for sand fraction. These results were comparable to the accuracy reported with previous studies in the region that employed geostatistics to predict soil properties (De Paz et al., 2011).

Altitude of the area above the sea level is quite low (maximum of 5.2 m), with a mean value of 2.5 m. This is really flat area, prone to inundation after intense precipitation events. These topographic characteristics may explain the heterogeneity of the topographic wetness index map (Fig. 4a), with a standard deviation much larger than the average value (3.6 vs. 1.3). Distance-to-geographic feature maps (Fig. 4b–h) were a wide set of explanatory variables of the DDT–DDE spatial distribution related to the hydrography, land cover, and infrastructures of the study area. These maps exhibited smooth surfaces, with average values ranging from a maximum of 5732 m for the distance to the Vinalopó River, located in the northeast extreme of the study area, to a minimum of 186 m for the distance to roads. Maximum distance to roads was 870 m, denoting a quite dense road network and high degree of human presence, as revealed by many sparse urban and industrial areas through all the zones (average distance to artificial land cover was about 700 m). Soil sealing by urban growth of high land capacity for agricultural use soils is a problem of major concern in this region (Navarro Pedreño et al., 2012).

### Application of machine learning techniques to assess the spatial distribution of DDT–DDE hotspots

The employment of machine learning techniques for acquiring knowledge about stream water pollution by pesticides is a feasible approach (Cordier et al., 2005). They allow the extraction of valuable information from large datasets that may be related to potential drivers/explanatory variables of the spatial distribution of these environmental pollutants. In this sense, DDT–DDE spatial distribution from our previous research was used as base information (Melendez-Pastor et al., 2021) devoted to identifying clusters of pesticides in the irrigation systems of an agricultural Mediterranean coastal floodplain between Vinalopó and Segura river courses. It should be taken into consideration that this persistent insecticide and its main metabolite (DDT–DDE) has not been used in the study area since the end of the past century in the 1970s (i.e., the Spanish Ministerial Order of 22 March, 1971 restricted the use of insecticides containing DDT due to their persistence and fat solubility). However, DDT–DDE is easily detected in the watercourses (detected in 91% of the water samples analyzed) and a high concentration cluster close to the artificial mouth of the Vinalopó River (confluence of the river within a drainage canal called “*Assarb de Dalt*”) had been identified.

RF machine learning technique to develop several experiments of supervised classification to assess the importance of several environmental factors on DDT–DDE spatial distribution was applied as it is a very interesting technique for our purpose because it does not overfit due to the Law of Large Numbers, thus acting as accurate classifier or regressor by injecting the right kind of randomness (Breiman, 2001). A categorization of the water samples (and subsequent values of the listed variables extracted at coincident locations) as belonging to a high–high cluster (hotspots) of DDT–DDE (category value = 1; *n* = 11), low–low clusters (cold spots) of DDT–DDE (category value = 2; *n* = 21), or non-significant cluster of DDT–DDE (category value = 0; *n* = 37) revealed an unbalance dataset of explanatory variables. In order to deal with unbalanced data, ensemble-based approaches are more adequate (Kulkarni et al., 2020). In this sense, RF machine learning technique was used as it is an ensemble-based classifier, good for unbalanced data (Tripathi et al., 2021).

Two different classification experiments were conducted, one with all the explanatory variables listed in Table 1 used for explaining the spatial distribution of DDT–DDE, and another one with a subset of variables previously selected with the mutual information technique. Continuous variables were normalized using a Min–Max normalization. Three different partitions of the database (i.e., training/validation) were also assessed: 70:30, 60:40, and 50:50.

**Fig. 5:**
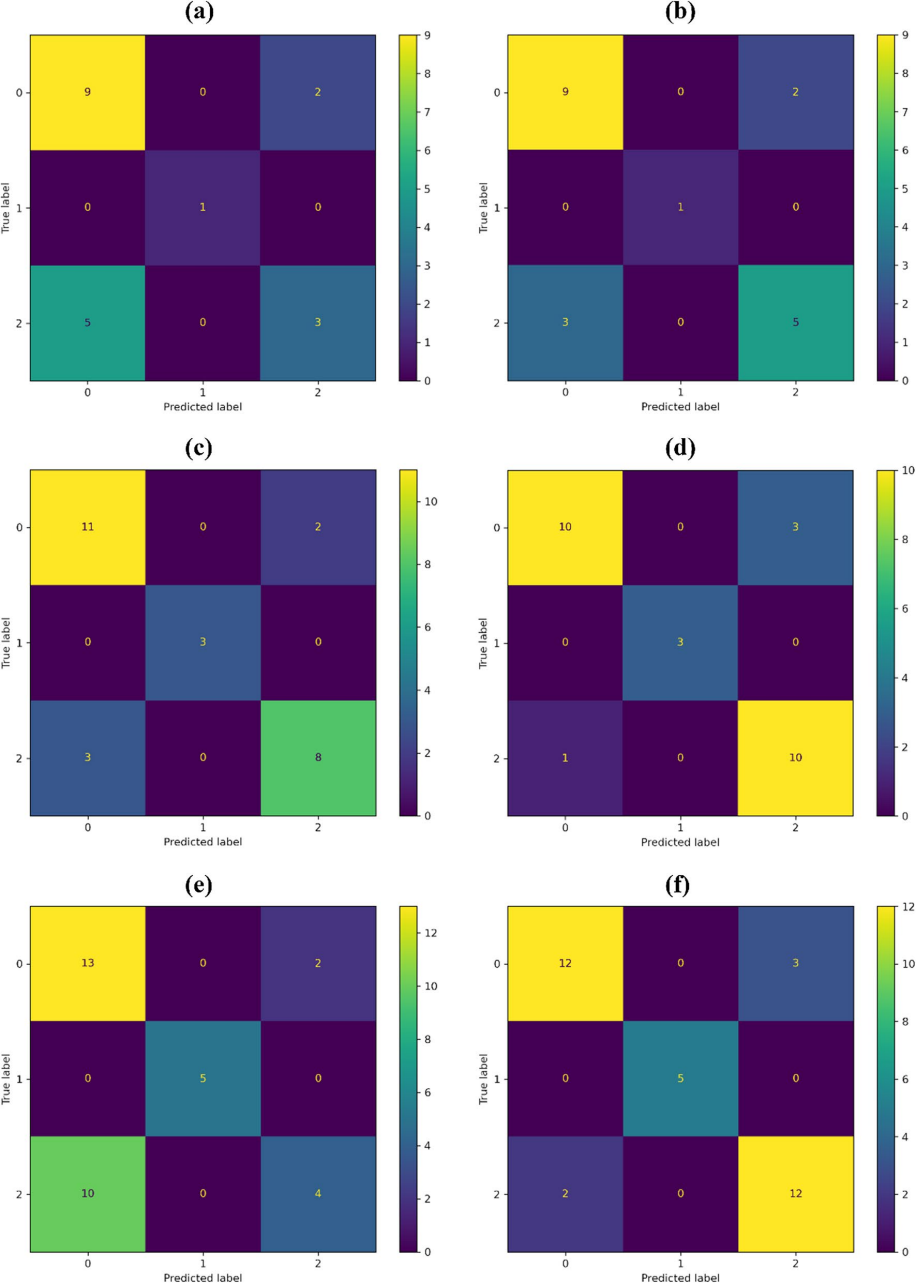
Error matrices of the classification models: **a** partition 70:30 for all predictive variables; **b** partition 70:30 for the mutual information subset; **c** partition 60:40 for all predictive variables; **d** partition 60:40 for the mutual information subset; **e** partition 50:50 for all predictive variables; and **f** partition 50:50 for the mutual information subset

For the application of RF with all explanatory variables, overall accuracy and kappa coefficient were higher for the training/validation ratio 60:40, with values of 0.815 and 0.685, respectively (Table 3). Average producer’s accuracy and user’s accuracy were also higher for the ratio 60:40, with values of 0.858 and 0.862, respectively. Additionally, error matrices (Fig. 5) are useful for a more detailed analysis of classification performance. For the three ratios of this first experimental stage (Fig. 5a, c, e), RF was highly efficient for predicting high-high clusters of DDT–DDE (class = 1). Classification mistakes could be mainly attributed to imprecise identification of samples belonging to category 2 (low-low clusters of DDT–DDE), that were classified as category 0 (non-significant cluster of DDT–DDE) in the predictions of the classifier (lower-left square in the error matrices). DDT–DDE reference cluster categories were different, but the pesticide concentration was similar.

**Table 3:** Accuracy assessment validation results for both datasets and the different training/validation partitions

Dataset	Partition	Overall accuracy	Kappa	Producer’s accuracy	User’s accuracy
All variables	70:30	0.650	0.317	0.731	0.748
	60:40	0.815	0.685	0.858	0.862
	50:50	0.647	0.419	0.717	0.744
MI selected variables	70:30	0.750	0.526	0.814	0.821
	60:40	0.852	0.751	0.893	0.893
	50:50	0.853	0.761	0.886	0.886

Overall accuracy and Cohen’s kappa are shown. Average values of producer’s and user’s accuracy for the three categories are also included

### Mutual information for explanatory variables selection

After the first experimental stage, mutual information was used to reduce the number of explanatory variables. MI was used to measure the amount of information that explanatory variables had about DDT–DDE values (Table 1). MI is zero if the two random variables are strictly independent (Kraskov et al., 2004). Oppositely, high MI values indicate a high relevance between the explanatory variable and DDT–DDE concentration.

**Fig. 6:**
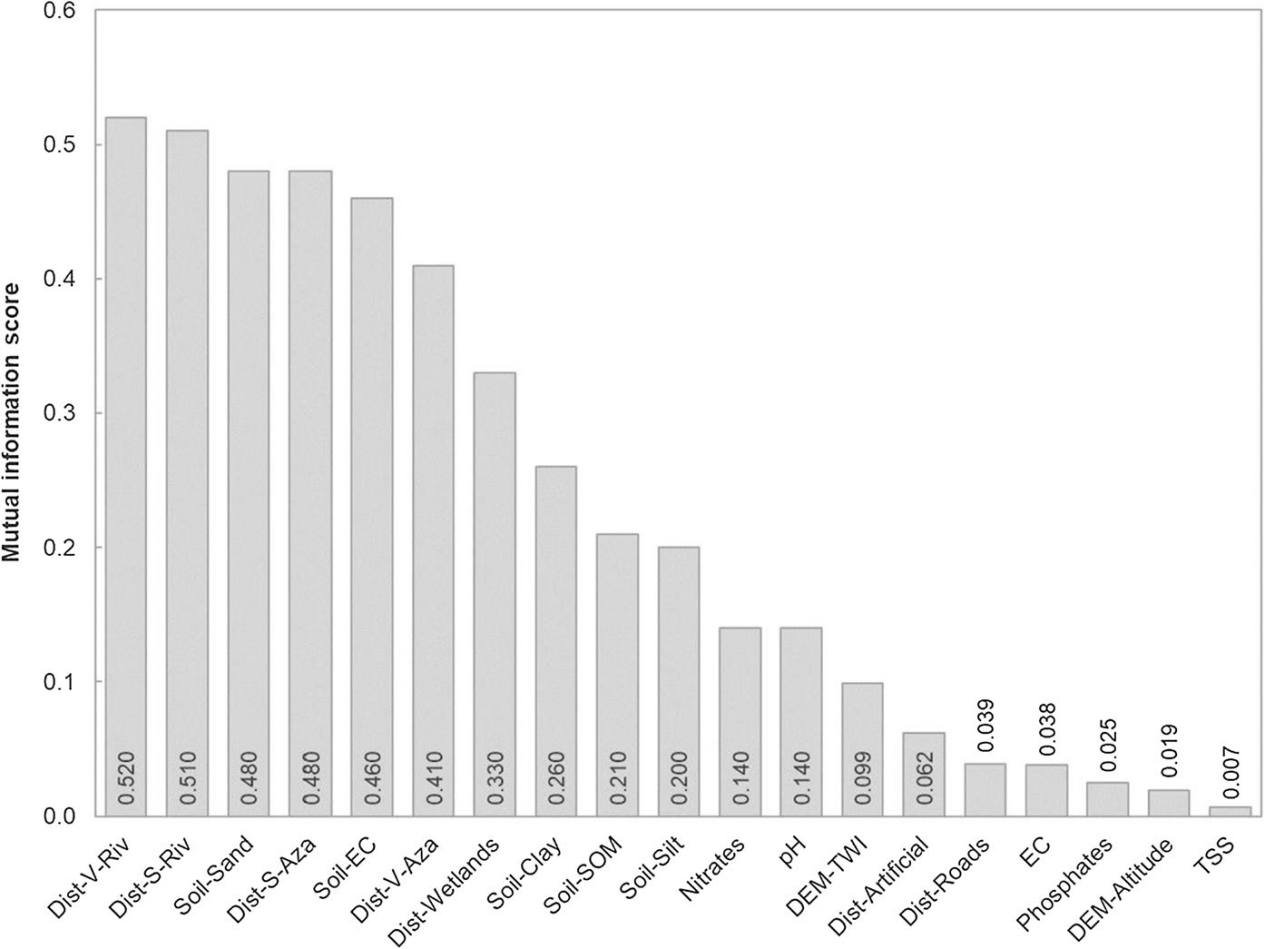
Scores obtained with the mutual information procedure for all predictive variables

The lowest MI value was obtained for total suspended solids (TSS) concentration of the water samples (MI = 0.007), and the highest MI value (0.52) was for the distance map from the Vinalopó river (Dist-V-Riv) (Fig. 6). As it can be observed in the bar plot, several variables had similar high MI values (MI > 0.4), then MI values decrease dramatically and stabilize for values lower than 0.005. In order to select the most appropriate variables for the RF classification, an iterative procedure was developed (Fig. 1) by choosing several explanatory variables with high MI values, applying RF classification, and computing accuracy assessment metrics.

The best performance was obtained with the first five explanatory variables (MI values higher than 0.45), namely: (1) distance to the Vinalopó river (Dist-V-Riv); (2) distance to the Segura river (Dist-S-Riv); (3) soil sand fraction (Soil-Sand); (4) distance to the drainage canals of the Segura river (Dis-S-Aza); and (5) soil electrical conductivity (Soil-EC).

Overall accuracy and kappa coefficient were higher for the training/validation ratio 50:50, with values of 0.853 and 0.761, respectively (Table 3). However, average producer’s accuracy and user’s accuracy were higher for the ratio 60:40, with average values of 0.893 and 0.893, respectively. For the three ratios of this second experimental stage (Fig. 5b, d, f), RF was highly efficient for predicting high-high clusters of DDT–DDE (class = 1). Respect to the first experimental stage, misclassification was notably reduced as observed by the higher average producer’s and user’s accuracy for the three ratios.

### Performance and utility of the proposed methodology

The use of MI for variables selection in machine learning experiments used to predict surface waters quality is a feasible technique. For example, Zhang et al. (2019) used MI for feature selection in a machine learning approach. They applied artificial neural networks for predicting water quality parameters in streams, concluding that the combination of MI and machine learning methods may be used to obtain more accurate results with a reduced number of explanatory variables. This reduction of the number of predictive variables is highly valuable in field research studies. The acquisition of information in the field is costly and time consuming, so a good option is to reduce the number of input variables in predictive models, if they are able to keep a high level of accuracy.

This research has evidenced the synergistic use of mutual information for optimizing RF classifications. Overall accuracy, kappa coefficient, and producer’s and user’s accuracy were very similar for the ratios 60:40 and 50:50 in the second experiment (variables selected with the MI procedure). For imbalanced datasets, some authors (Tripathi et al., 2021) suggest the importance of taking into account true positive cases, quantified by the sensitivity metric (equivalent to producer’s accuracy in multiclass error matrices), as the important metric to be used in performance evaluation. Producer’s accuracy of category 1 (hotspots of DDT–DDE) was one for both partitions, since no features were incorrectly classified. Producer’s accuracy of category 2 (low-low clusters of DDT–DDE) was slightly better for the ratio 60:40 (0.909 for 60:40 vs. 0.857 for 50:50). Producer’s accuracy of category 0 (non-significant points) was slightly better for the ratio 50:50 (0.769 for 60:40 vs. 0.800 for 50:50). Considering the sensitivity metric for both classification approaches, the ratio 60:40 could be more suitable for detecting low-low clusters of DDT–DDE.

Respect to the variables that may contribute to explain the current distribution of DDT–DDE in the watercourses of the study area, we found two kind of variables selected by the MI procedure:

1. Distance variables to geographical features, including distance to the Vinalopó river, to the Segura river, and to the drainage canals of the Segura river. The location of the high-high cluster of DDT–DDE close to the artificial mouth of the Vinalopó river, and its relationship with the Vinalopó river watercourse is evidenced by the highest MI value (0.520) for the variable distance to the Vinalopó river (Dist-V-Riv). This relationship is direct, because as closer to the artificial mouth of the Vinalopó river, as higher the probability to find the high-high cluster of DDT–DDE. The relationship among the variable distance to the Vinalopó river, the variables’ distance to the Segura river (Dist-S-Riv), and distance to the drainage canals of the Segura river (Dis-S-Aza) is inverse. As closer to the Segura river systems, as lower the probability to find the high-high cluster of DDT–DDE. In fact, average DDT–DDE concentration in the watercourse and drainage canals of the Segura river is 1.19 μg/L, while the concentration for the watercourse and drainage canals of the Vinalopó river is 1.93 μg/L. This variable associated to the location of areas with high values of DDT–DDE attributable to past use of the pesticide. This region of southeast Spain had endemic malaria until the 1960s (Bueno & Jiménez, 2008), and the presence of large wetlands, irrigation water reservoirs, and irrigation and drainage canals favored the *Anopheles* mosquitoes, that were combated with an intensive use of insecticides (especially DDT) in the past. The current presence of hotspots of DDT–DDE in surface waters is frequent in areas where malaria was endemic and extensive history of DDT application has left a permanent mark on the environment (Horak et al., 2021).
2. Soil properties, including soil electrical conductivity and soil sand fraction. Previous works have shown that soils of that area have high salinity with a clear north-to-south spatial pattern of decreasing values (Bas-Niñerola et al., 2017). Underlying causes of the spatial distribution of soil salinity should be found in the processes of salinization that affect this semiarid Mediterranean coastal area. Secondary salinization of the soil and remote transport of soluble salts (Schofield et al., 2001; Szabolcs & Fink, 1974) through the Vinalopó River may explain the spatial pattern of soil salinity in the study area. Additionally, particle size distribution is one of the most important soil attributes that largely determines soil hydraulic behavior and water storage, handling characteristics under tillage, and susceptibility to degradation (Rawlins et al., 2009). Respect to the soil texture, root zones generally act as a primary environmental sink for pesticides, but sand-rich profiles are less effective because of their low chemical adsorption capacity and high hydraulic conductivity (Lee et al., 2010). Higher hydraulic conductivity of soils with a larger abundance of sand fraction is expected and may explain the higher concentrations of DDT–DDE in the surface waters as a result of previous leaching of the pesticide from its reservoirs.

## Conclusions

This study has advanced in understanding the presence of detectable concentrations of a supposedly obsolete pesticide in drainage waters of a Mediterranean flooded coastal area, giving also an adequate explanation through the application of a procedure for the identification of clusters of high (or low) concentration of DDT–DDE by using a set of environmental variables and a combination of machine learning techniques.

The RF classifier was useful to deal with datasets of water and soil characteristics and topographic and geographical distance, as it has been demonstrated. In addition, the employment of an information selection algorithm such as Mutual Information for the iterative optimization of the classification is a promising tool to refine the number of environmental variables to be acquired during field surveys, with the subsequent optimization of the analytical procedures and the research process to explain and follow the pollution of the waters.

Specifically, for our study case, several hydrological and soil variables were the most important for explaining the spatial distribution of DDT–DDE concentrations. Pesticide input was related with the Vinalopó river watercourse, that also generates a notable flux of soluble salts as denoted by the high electrical conductivity, confirmed by the saline soils presented around the artificial mouth of the river. DDT–DDE transport seems to be easy through the sandy soils (coarse texture), located around the mouth of the Vinalopó river. Both parameters, salinity and coarse texture, and the distance to the watercourses were the most important variables that explained the presence of pesticides in drainage waters.

## Data Availability

Data available on request from the authors.
